# Magnitude of the Digital Placebo Effect and Its Moderators on Generalized Anxiety Symptoms: Systematic Review and Meta-Analysis

**DOI:** 10.2196/74905

**Published:** 2025-07-31

**Authors:** Takashi Hosono, Rinka Tsutsumi, Yuki Niwa, Masuo Kondoh

**Affiliations:** 1 Graduate School of Pharmaceutical Sciences, The University of Osaka, 1-6 Yamadaoka, Suita, Osaka, 5650871, Japan; 2 Clinical Research, R&D, NS Pharma Inc., Paramus, NJ, United States; 3 Center for Infectious Disease Education and Research (CiDER), The University of Osaka, Osaka, Japan

**Keywords:** digital health, digital intervention, digital therapeutics, randomized clinical trial, psychiatry, psychiatric, generalized anxiety disorder

## Abstract

**Background:**

Digital therapeutics (DTx) have attracted attention as the substitutes or add-ons to conventional pharmacotherapy. The number of clinical trials for DTx has increased recently, and one of the main targets for DTx is psychiatric disorders. Generalized anxiety disorder (GAD) is one of the most common and notable psychiatric disorders, and it’s known that the magnitude of placebo effect in the pharmacotherapy is quite large. The randomized controlled trials (RCTs) with digital placebos are the most reliable clinical trials to evaluate the safety and efficacy of DTx. However, the magnitude of the digital placebo effect and its moderators on GAD have not been investigated, although they are critical to assess the true treatment effect of DTx.

**Objective:**

The objectives of this study were to identify RCTs with digital placebos as comparators that evaluated GAD assessment scores, to review the characteristics of the RCTs and of the digital placebos in the systematic review, and to investigate the magnitude and its moderators in the meta-analysis.

**Methods:**

The RCTs evaluating the GAD assessment scores by setting digital placebos as comparators were identified by searching the database of PubMed, Web of Science, and Scopus in July 2024. The characteristics of the RCTs and of the digital placebos were reviewed systematically. The meta-analysis, including subgroup analyses and meta-regressions, was conducted to investigate the magnitude and its moderators of the digital placebos.

**Results:**

A total of 54 RCTs were included in the systematic review and 32 RCTs with 3 GAD assessment scores were included in the meta-analysis with a total of 5311 participants. The magnitude of digital placebos for all the included studies was small to moderate (Hedges *g*=0.28, 95% CI 0.18-0.38). The subgroup analyses showed the significant difference in the magnitude among target population (*P*=.03), placebo approach (*P*=.02), and baseline values (*P*=.02). The meta-regressions also indicated that the primary psychiatric patients in the target population (*P*=.01), “Removed” type in placebo approach (*P*=.04) and high baseline values (*P* =.02) were moderators for the magnitude of digital placebos.

**Conclusions:**

This study showed the small-to-moderate and statistically significant digital placebo effect on GAD assessment scores. Target population, placebo approach, and baseline values were also identified as the moderators of the placebo effect. It would be effective to create the study protocols for the DTx trials with digital placebos by considering the moderators identified in this study.

## Introduction

Digital Therapeutics (DTx) are rapidly spreading as substitutes or add-ons to conventional pharmacotherapy recently. According to the definition by International Organization for Standardization, DTx are defined as health software intended to treat or alleviate a disease, disorder, condition, or injury by generating and delivering a medical intervention that has a demonstrable positive therapeutic impact on a patient [1]. The main difference between DTx and conventional wellness apps is that DTx are developed with clinical evidence usually generated from clinical trials [2].

In many countries, DTx are regulated through the similar regulatory framework with medical devices as part of software as a medical device (SaMD), which is defined as software intended to be used for one or more medical purpose(s) that perform(s) these purposes without being part of hardware medical device by International Medical Device Regulators Forum [3]. Some regulatory authorities have recently implemented the policies that promote the development and the patient access of DTx and SaMD [4].

With the implementation of regulatory policies, the number of clinical trials of DTx is also increasing in the world [5]. The designs of the confirmatory studies of DTx are basically comparable to those of drugs and medical devices except for some elements such as blinding and comparators. Traditionally, no intervention, waiting list, or treatment-as-usual was widely used as the comparators in the clinical trials of DTx due to the technical difficulties to keep blinding by using appropriate comparators [6].

However, with the advancement of digital technology, digital placebos have been recently adopted in the clinical trials of DTx to evaluate the true effect of active interventions in blind manners appropriately [7]. Digital placebos are also called digital shams, sham apps, etc, in contexts, but the international standardized definition of digital placebos has never been fully discussed. It is critical to set appropriate digital placebos as comparators and manage them accordingly in the clinical trials to assess the true treatment effect of DTx, but there are still some hurdles related to the validation, the design variability, the patient engagement, the infrequent reports of the results, etc [8].

The therapeutic areas of DTx are diverse, but psychiatric disorders are one of the major targets because of the benefit from cognitive behavioral interventions [9]. In the *International Classification of Diseases 11*, the clinical trials of DTx for mental, behavioral, and neurodevelopmental disorders have been mostly conducted recently [5]. In addition to depression, substance use disorders, posttraumatic stress disorders, etc, anxiety disorders are one of the main and notable psychiatric disorders, affecting 301 million people in the world in 2019 [10]. The World Health Organization also indicated that the COVID-19 pandemic triggered 25 % increase in prevalence of anxiety and depression worldwide [11].

Anxiety disorders are classified into generalized anxiety disorder (GAD), social anxiety disorder (SAD), panic disorder, agoraphobia etc. GAD is defined as excessive anxiety and worry about a number of events or activities, occurring more days than not for at least 6 months [12]. Psychotherapy, such as cognitive behavioral therapy, as well as pharmacotherapy, is widely used as the first-line treatment for anxiety disorders, including GAD, in the various treatment guidelines [13-15]. The disadvantages of face-to-face psychotherapy are low accessibility and high cost. Alternatively, the digital interventions, including DTx, have gained attention for the treatment of psychiatric disorders to overcome the hurdles [9].

The effect of digital interventions for anxiety, including GAD, has been investigated in the systematic review and the meta-analysis [16]. However, the magnitude and its moderators of digital placebos on GAD have not been investigated. The magnitude of placebo effect is quite large in GAD [17], and it is critical to identify the magnitude and its moderators of digital placebos and to reflect them to the protocols of clinical trials appropriately.

The objectives of this study were to identify the randomized controlled trials (RCTs), which used digital placebos as comparators, to review the characteristics of the RCTs and of the digital placebos systematically, and to investigate the magnitude and its moderators on GAD assessment scores in the meta-analysis. To our knowledge, this is the first research to investigate the magnitude and its moderators of digital placebos.

## Methods

### Search Strategy

This study followed the Preferred Reporting Items for Systematic Reviews and Meta-Analyses (PRISMA) reporting guidelines [18] (see Checklist 1). In terms of the PICO (Population, Intervention, Control, and Outcome) framework, the target population of this study was adults aged 18 years and older, but was not limited to GAD diagnosed patients to generate a broader data pool. Instead, the impact of difference among the target population was investigated with the subgroup analysis in the meta-analysis. The intervention and the control were limited to DTx as active interventions and to digital placebos as controls, respectively. The study designs were also limited to RCTs. As for the outcome, GAD assessment scores were adopted in this study.

The digital placebos were defined based on previous research in this study [8]. In brief, digital placebos are comparators designed to mimic the DTx (eg, with a similar design, components, and duration of treatment as the DTx), but the DTx active principle or component being removed or reduced in intensity. The control with a different delivery type from the intervention was not regarded as the digital placebo in this study. For example, delivering control via the web while providing the intervention through virtual reality was not considered as a digital placebo. We included the studies with DTx that were not intended to treat patients in our study if the GAD assessment scores were adopted because the target population in this study was not limited to GAD diagnosed patients.

The papers reporting the results of clinical trials that used digital placebos as comparators were identified in the following way. The total of 3 databases, including PubMed, Web of Science, and Scopus, were systematically searched with the keywords: Anxiety AND (random* OR RCT) AND (blind OR blinded) AND (digital OR mhealth OR ehealth OR app OR apps OR application* OR smartphone OR mobile OR online OR computer-based OR web-based OR internet-based OR internet-delivered OR “virtual reality ” OR VR OR “ augmented reality ” OR AR OR wearable* OR game* OR gamifi*), in July 2024. Multimedia Appendix 1 shows the detailed search strategy . The search language was limited to English.

After excluding the duplicates and the papers before 2000 automatically on the reference management software, the papers on nonclinical trials, drugs, and supplements, the ones without results, and the ones with hardware evaluation ( eg, transcranial direct current stimulation and acupuncture) were excluded by the review of the titles and the abstracts of each report.

Next, the papers without digital placebos as comparators were excluded with the full-text review. The trials which adopted waiting list or treatment-as-usual without any digital interventions were excluded in this process. Subsequently, we excluded the papers from the duplicated studies and the ones for children or adolescents aged 18 years and younger. We also excluded the papers that did not set any GAD assessment scores as the primary or secondary endpoints. Those included the reports which adopted only the scores for SAD and the ones for children or adolescent anxiety. The studies without enough data for statistical analysis, the ones with wrong study designs to evaluate the effect of digital placebos, and the ones with less than 5 pre or post data were excluded. In order to control heterogeneity, the meta-analysis was conducted only with 3 GAD assessment scores, the reported number of which were the largest. The identification of the target papers in this study was conducted by TH and RT independently and the discrepancies were resolved through consensus meetings.

### Data Extraction

The following information and variables were extracted from the selected papers for this systematic review and meta-analysis: (1) general information (author, clinical trial registration number, and publication year), (2) demographic characteristics (target population and age), (3) placebo device characteristics (placebo delivery type and placebo approach), (4) study design information (number of total patients, blindness, treatment period, number of group, and country), and (5) outcome information (primary and secondary endpoints, mean (SD) of pre and post intervention of comparators).

The target population was classified into 3 groups in this study. The group of primary psychiatric disorders included the participants who had been diagnosed as psychiatric disorders or who met the criteria of specific psychiatric disorders based on clinical guidelines such as *Diagnostic and Statistical Manual of Mental Disorders, Fifth Edition* at the timing of screening [12]. The group of other diseases included the patients with underlying conditions rather than psychiatric disorders such as cancers and pains. The others were classified into the group of nonpatients.

The delivery type and approach of digital placebos was also set based on previous research [8]. The delivery type included “Web”, “Mobile”, “Computer”, “Virtual Reality (VR) / Augmented Reality (AR) ” and “Wearables”.The placebo approach was classified into the following 4 categories. “Replaced” is an approach which replaces the active component with an inactive or neutral component. “Removed” is an approach which simply removes active component. “Unrelated” is an approach that the active component is replaced by a different active component, which is unrelated. “Less” is an approach that is less intense version of the active component.

Only 1 GAD assessment score was adopted from 1 study. The GAD assessment score in primary endpoint was prioritized if any. If no GAD assessment score was set in primary endpoint and some secondary endpoints for GAD were set in a study, we adopted the endpoint with the smallest placebo effect conservatively. The postintervention data at the end of treatment or the closest were adopted in each study although the long-term effect after the completion of the treatment was investigated in some selected studies.

If the means or the SDs of the GAD assessment scores for meta-analysis were not reported in the papers, the corresponding author was contacted by email. If no reply was obtained, we imputed the data as recommended by the Cochrane group or extracted the data from the graphs in the papers, if possible [19]. The studies without enough information for meta-analysis, regardless of the work above, were excluded from this study. Sensitivity analysis was conducted by excluding the paper that required imputation for the means or the SDs. The data extraction from the identified papers was conducted by TH and double-checked by RT. The discrepancies were resolved through consensus meetings.

### Statistical Analysis

The statistical analyses were performed by using R (version 4.4.2; R Foundation for Statistical Computing). The magnitude of placebo effect was computed based on the sample sizes, the means and the SDs of the baseline and the postintervention of the comparator group in each study. We adopted the Hedges *g* to estimate the magnitude of digital placebo effect because the studies with small sample size were included in this study. A random effects model was used by assuming high heterogeneity. The publication bias was evaluated by the Begg modified funnel plot, the Duval and Tweedie trim-and-fill procedure, and the Egger regression intercept.

Subgroup analyses and meta-regressions were conducted to explore the reasons for heterogeneity and the potential moderators for the magnitude of digital placebo effect by using variables which were extracted from the selected studies. Variables were considered statistically significant when the *P* values were under .05 in both the subgroup analyses and the meta-regressions.

Risk of bias assessment was performed by using the revised Cochrane risk-of-bias tool for randomized trials [19]. Sensitivity analysis was conducted by excluding the studies with high risk of bias.

## Results

### Study Selection for Systematic Review

**Figure 1.**
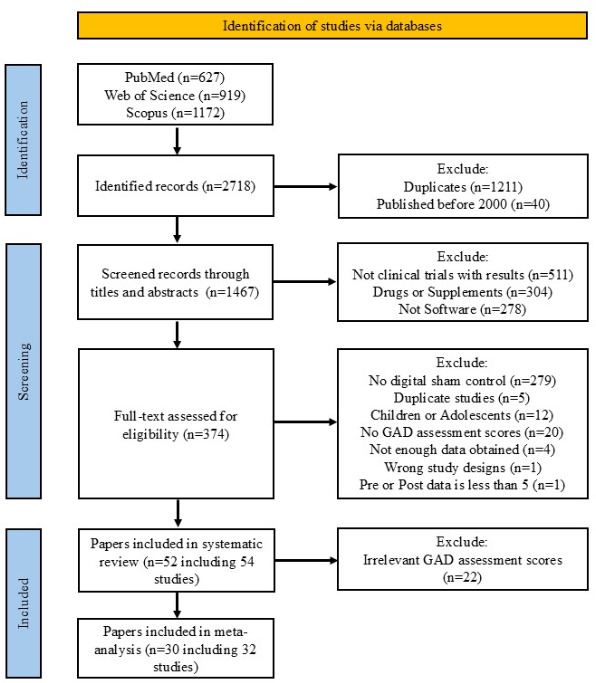
PRISMA (Preferred Reporting Items for Systematic Reviews and Meta-Analyses) chart. The PRISMA chart for systematic review and meta-analysis is shown with the number of records included or excluded at each step. GAD: generalized anxiety disorder.

The PRISMA flowchart (see Figure 1) shows that 2718 records were identified as potentially relevant. Following screening with titles, abstracts, and methods, 374 papers were identified. Through the full-text reviews, 52 papers and 54 studies were identified for the systematic review [20-71]. 1 paper included 2 studies [21] and the population in another article was divided into 2 groups in accordance with the mental risk they defined. We dealt with those as independent studies [25].

### Characteristics of the Included Studies for Systematic Review

Table 1 lists the studies included in this systematic review. Multimedia Appendix 2 summarizes the characteristics of the studies. All the selected studies were randomized, blinded, parallel group comparison studies, except for one in which cross-over design was adopted. Almost 65 % (35/54) of the studies were published in 2019 and after. Target population was almost evenly divided into 3 groups. The placebo delivery type in most included studies was mobile or web. Most of the placebo approach was that the active component was replaced with inactive or neutral components. The total numbers of participants in the selected studies were relatively large and approximately 15% (8/54) of the selected studies included over 501 participants. Almost half of the studies were double-blind studies. The most frequent treatment period and the number of groups was >31 days and <90 days and 2 groups, respectively. The multicountry clinical trials were conducted only in 3 studies and none of them were multiregional clinical trials (MRCTs).

**Table 1.** The studies included in the systematic review.

Study, year	Target population	Age (years), mean (SD)	Placebo delivery	Placebo approach	Total patients, N	Blindness	Treatment (days)	Groups, n	Countries	Endpoint
Carlbring et al [20], 2012	Primary	38.0 (12.0)	Web	Replaced	79	Double	28	2	Single	BAI ^a^
Sharpe et al Study 1 [21], 2012	Other diseases	40.6 (15.8)	Web	Replaced	34	Double	1	2	Single	DASS-A^b^
Sharpe et al Study 2 [21], 2012	Other diseases	45.6 (14.5)	Web	Replaced	34	Double	28	2	Single	DASS-A^b^
Boettcher et al [22], 2013	Primary	42.8 (11.5)	Web	Replaced	129	Double	14	6	Single	BAI^a^
Glozier et al [23], 2013	Other diseases	58.4 (6.6)	Web	Replaced	562	Double	84	2	Single	GAD-7^c^
Enock et al [24], 2014	Nonpatients	34.8 (11.4)	Mobile	Replaced	429	Double	28	3	Single	PSWQ^d^
Musiat et al High [25], 2014	Nonpatients	21^e^	Web	Unrelated	181	Single-P^r^	84	2	Single	GAD-7^c^
Musiat et al Low [25], 2014	Nonpatients	21^e^	Web	Unrelated	859	Single-P	84	2	Single	GAD-7^c^
Salemink et al [26], 2014	Primary	38.6 (10.8)	Web	Replaced	47	Double	11	2	Single	STAI-ST^g^
Taylor-Rodgers and Batterham [27], 2014	Nonpatients	21.9 (1.9)	Web	Unrelated	67	Single-P	21	2	Single	GAD-7^c^
Green et al [28], 2014	Other diseases	59.6 (13.4)	Web	Replaced	200	Single-P	1	2	Single	STAI-ST^g^
Buntrock et al [29], 2016	Nonpatients	44.4 (11.8)	Web	Replaced	406	Single-I^h^	3 ‐ 6 weeks	2	Single	HADS-A^i^
Carleton et al [30], 2017	Primary	39.7 (12.5)	Web	Replaced	113	Double	28	2	Single	STAI-TR^j^
Dennis-Tiwary et al [31], 2017	Nonpatients	31.1 (6.2)	Mobile	Replaced	33	Double	28	2	Single	DASS-A^b^
McAllister et al [32], 2017	Primary	39.7 (16.0)	Web	Replaced	31	Double	28	2	Single	STAI^k^
Peters et al [33], 2017	Nonpatients	24.9 (6.3)	Web	Replaced	111	Double	42	2	Single	STAI-ST^g^
Baker et al [34], 2018	Other diseases	42.0 (15.4)	Web	Replaced	39	Single-P	56	2	Single	BAI^a^
Bennell et al [35], 2018	Other diseases	61.3 (7.1)	Web	Replaced	144	Double	56	2	Single	DASS-A^b^
Kyrios et al [36], 2018	Primary	34.2 (9.9)	Web	Replaced	179	Double	84	2	Single	HAM-A^l^
Amir et al [37], 2019	Primary	35.3 (12.0)	Computer	Removed	169	Double	84	4	Single	HAM-A^l^
Clarke et al [38], 2019	Other diseases	57.7 (10.0)	Web	Removed	780	Double	84	2	Single	GAD-7^c^
Glozier et al [39], 2019	Primary	58.1 (6.1)	Web	Replaced	87	Double	84	2	Single	HAM-A^l^
Ahorsu et al [40], 2020	Other diseases	38.0 (9.9)	Mobile	Replaced	320	Double	42	2	Single	HADS-A^i^
Fuller-Tyszkiewicz et al [41], 2020	Nonpatients	39.2 (5.9)	Mobile	Removed	183	Single-P	35	2	Single	DASS-A^b^
Majd et al [42], 2020	Primary	35.3 (5.8)	Mobile	Replaced	312	Single-I	42	3	Single	HADS-A^i^
Andersson et al [43], 2021	Primary	43.0 (14.0)	Web	Replaced	311	Single-P	70	2	Single	PSWQ^d^
Bove et al [44], 2021	Other diseases	49.2 (10.9)	Mobile	Replaced	44	Double	42	2	Single	STAI-TR^j^
Fiol-DeRoque et al [45], 2021	Nonpatients	40.6 (9.6)	Mobile	Replaced	482	Single-P	14	2	Single	DASS-A^b^
Hirsch et al [46], 2021	Primary	35.7 (11.5)	Web	Replaced	230	Single-P	28	2	Single	PSWQ^d^
Morriss et al [47], 2021	Nonpatients	38.4 (14.3)	Web	Replaced	790	Single-I	42	2	Single	GAD-7^c^
Ong et al [48], 2021	Other diseases	58.0 (16.0)	Mobile	Replaced	182	Single-I	365	2	Single	HADS-A^i^
Aganov et al [49], 2022	Nonpatients	39.1 (9.4)	VR^m^	Replaced	94	Single-I	7	2	Single	STAI-ST^g^
Cuijpers et al [50], 2022	Primary	27.1 (8.1)	Web	Replaced	680	Single-P	56	2	Single	GAD-7^c^
Haas et al [51], 2022	Primary	36.9 (13.2)	Web	Replaced	161	Single-I	35	3	Single	BAI^a^
Rodrigues et al [52], 2022	Other diseases	48.5 (16.9)	VR	Replaced	44	Double	1	2	Single	HADS^n^
Teles et al [53], 2022	Nonpatients	58.8 (12.4)	Web	Replaced	42	Single-I	84	2	Single	HADS-A^i^
Torok et al [54], 2022	Nonpatients	21.7 (2.2)	Mobile	Replaced	455	Double	42	2	Single	GAD-7^c^
Ditton et al [55], 2023	Nonpatients	25.4 (5.8)	Mobile	Less	108	Double	35	3	Single	DASS-A^b^
Eek et al [56], 2023	Primary	53.7 (10.7)	Web	Less	166	Single-P	84	3	Single	GAD-7^c^
Ehlers et al [57], 2023	Primary	35.8 (11.5)	Web	Unrelated	217	Single-I	84	3	Single	GAD-7^c^
Fatori et al [58], 2023	Primary	32.3 (4.9)	Mobile	Replaced	81	Double	56	2	Single	GAD-7^c^
Hoffmann et al [59], 2023	Nonpatients	47.0 (13.0)	Web	Removed	89	Double	30	2	Multiple	HADS-A^i^
Karlsson-Good et al [60], 2023	Primary	39.3 (14.1)	Web	Less	273	Single-P	84	2	Single	BAI^a^
Nijman et al [61], 2023	Primary	39.7 (12.4)	VR	Unrelated	81	Single-I	56	2	Single	BAI^a^
Sharpe et al [62], 2023	Other diseases	49.9 (13.9)	Web	Replaced	288	Double	28	4	Single	DASS-A^b^
van Gelder et al [63], 2023	Nonpatients	35.1 (NA)	Web	Replaced	198	Single-P	90	2	Single	HADS-A^i^
Zion et al [64], 2023	Other diseases	52.5 (11.4)	Mobile	Replaced	449	Double	84	2	Single	PROMIS-A^o^
Kleinau et al [65], 2024	Nonpatients	24^e^	Mobile	Replaced	1493	Single-I	56	2	Single	GAD-7^c^
Romano et al [66], 2024	Other diseases	34.0 (10.2)	Web	Replaced	62	Single-P	56	2	Multiple	DASS-A^b^
Sandhu et al [67], 2024	Nonpatients	30.3 (5.1)	Web	Removed	306	Single-P	365	2	Single	DASS-A^b^
Scazufca et al [68], 2024	Primary	60^p^	Mobile	Replaced	603	Single-I	42	2	Single	GAD-7^c^
Thompson et al [69], 2024	Primary	33.0 (1.4)	Web	Unrelated	133	Single-P	42	2	Multiple	DASS-A^b^
Vereschagin et al [70], 2024	Nonpatients	20^e^	Mobile	Removed	1489	Single-I	30	2	Single	GAD-7^c^
Zainal et al [71], 2024	Primary	21.8 (3.4)	Mobile	Removed	191	Single-I	14	2	Single	GADQ^q^

a BAI: Beck Anxiety Inventory.

b DASS-A: Depression Anxiety Stress Scale-Anxiety.

c GAD-7: Generalized Anxiety Disorder-7.

d PSWQ: Penn State Worry Questionnaire.

e Median (no information for IQR value was described in the paper).

f Single-P: single blinding against patients.

g STAI-ST: State Trait Anxiety Inventory-State.

h Single-I: single blinding against investigators.

i HADS-A: Hospital Anxiety and Depression Scale-Anxiety.

j STAI-TR: State Trait Anxiety Inventory-Trait.

k STAI: State Trait Anxiety Inventory.

l HAM-A: Hamilton Anxiety Rating Scale.

m VR: virtual reality.

n HADS: Hospital Anxiety and Depression Scale.

o PROMIS-A: Patient-Reported Outcomes Measurement Information System-Anxiety.

p Target age 60-70 years.

q GADQ: Generalized Anxiety Disorder Questionnaire.

### Study Selection for Meta-Analysis

The 3 most frequently adopted GAD assessment scores in the selected studies for this meta-analysis were Generalized Anxiety Disorder-7 (GAD-7), Depression Anxiety Stress Scales-Anxiety (DASS-A), and Hospital Anxiety and Depression Scale-Anxiety (HADS-A; see Multimedia Appendix 2) . These 3 assessment scores covered 74.9% (5311/7092) of all cases included in the systematic review. The brief descriptions for each assessment score are as follows.

GAD-7 is a 7-item self-reported scale to assess anxiety symptoms over the past 2 weeks. Scores range from 0 (not to all) to 3 (nearly every day), with a maximum score of 21 [72]. DASS-A is a 21-item self-reported scale to assess depression, anxiety, and stress. Items are rated on 4-point scale ranging from 0 (did not apply to me at all) to 3 (applied to me very much). DASS-A is the anxiety component of DASS [73]. HADS-A is a 14-item self-reported scale that assesses anxiety (7 items) and depression (7 items). Scores of each item range from 0 to 3 with higher score representing higher levels of anxiety and depression. HADS-A is the anxiety component of the Hospital Anxiety and Depression Scale [74]. All of them are self-reported scales that assess anxiety and several studies reported high correlation among GAD-7, DASS-A, and HADS-A in specific diseases and population [75,76].

### Magnitude of Digital Placebo Effect

**Figure 2.**
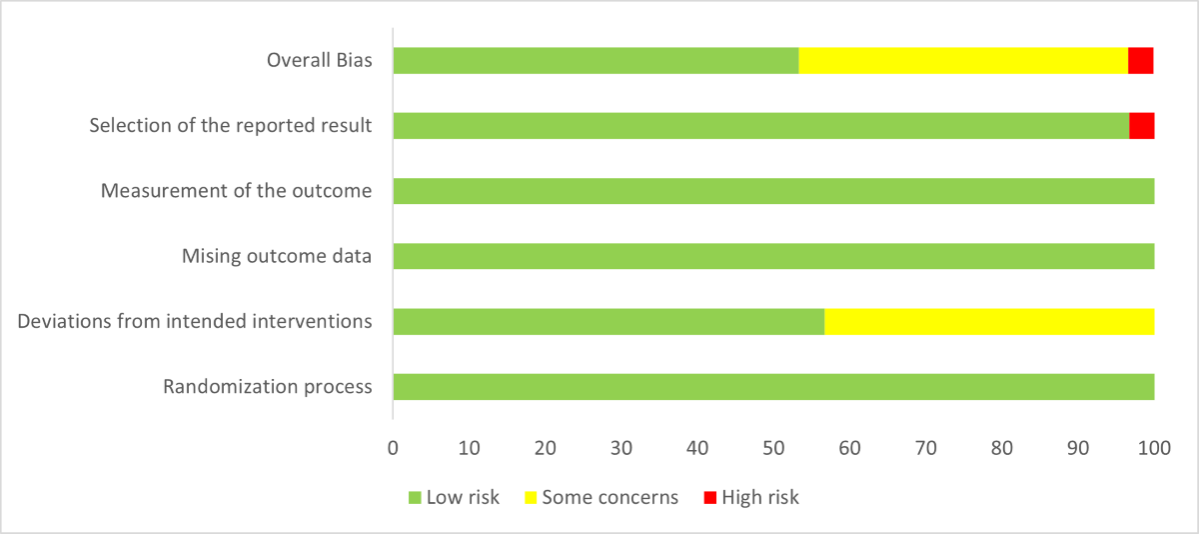
Results of risk of bias assessment of included studies for meta-analysis using the revised Cochrane risk-of-bias tool for randomized trials [19].

A total of 32 studies, which adopted GAD-7, DASS-A, or HADS-A as assessment scores were included in the meta-analysis with the total number of 5311 [21,23,25,27,29,31,35,38,40-42,45,47,48,50,53-59,62,63,65-70]. The pooled effect size for all included studies was Hedges *g*=0.28 (95% CI 0.18-0.38) with an overall *I*^2^ = 76%. The Egger test did not indicate publication bias (*P* =.47). We also investigated the risk of publication bias with a funnel plot visually and applied the trim-and-fill method to investigate any asymmetry of the funnel plot. The funnel plot did not show any substantial publication bias and the trim-and-fill analysis showed the presence of 2 unpublished studies (see Multimedia Appendix 3). Considering these studies in the pooled analysis, the magnitude of placebo effect was adjusted to Hedges *g*=0.24 (95% CI 0.13-0.35). Figure 2 shows the summary the results of risk of bias and Multimedia Appendix 4 shows the detailed results in each study . The placebo effect remained when considering only studies with a low risk of bias (Hedges *g*=0.26, 95% CI 0.15-0.36). The sensitivity analysis by excluding the studies that required imputation of means or SDs did not show any significant changes on the outcome (Hedges *g* =0.30, 95% CI 0.20-0.40).

### Results of Subgroup Analyses and Meta-Regressions

**Figure 3.**
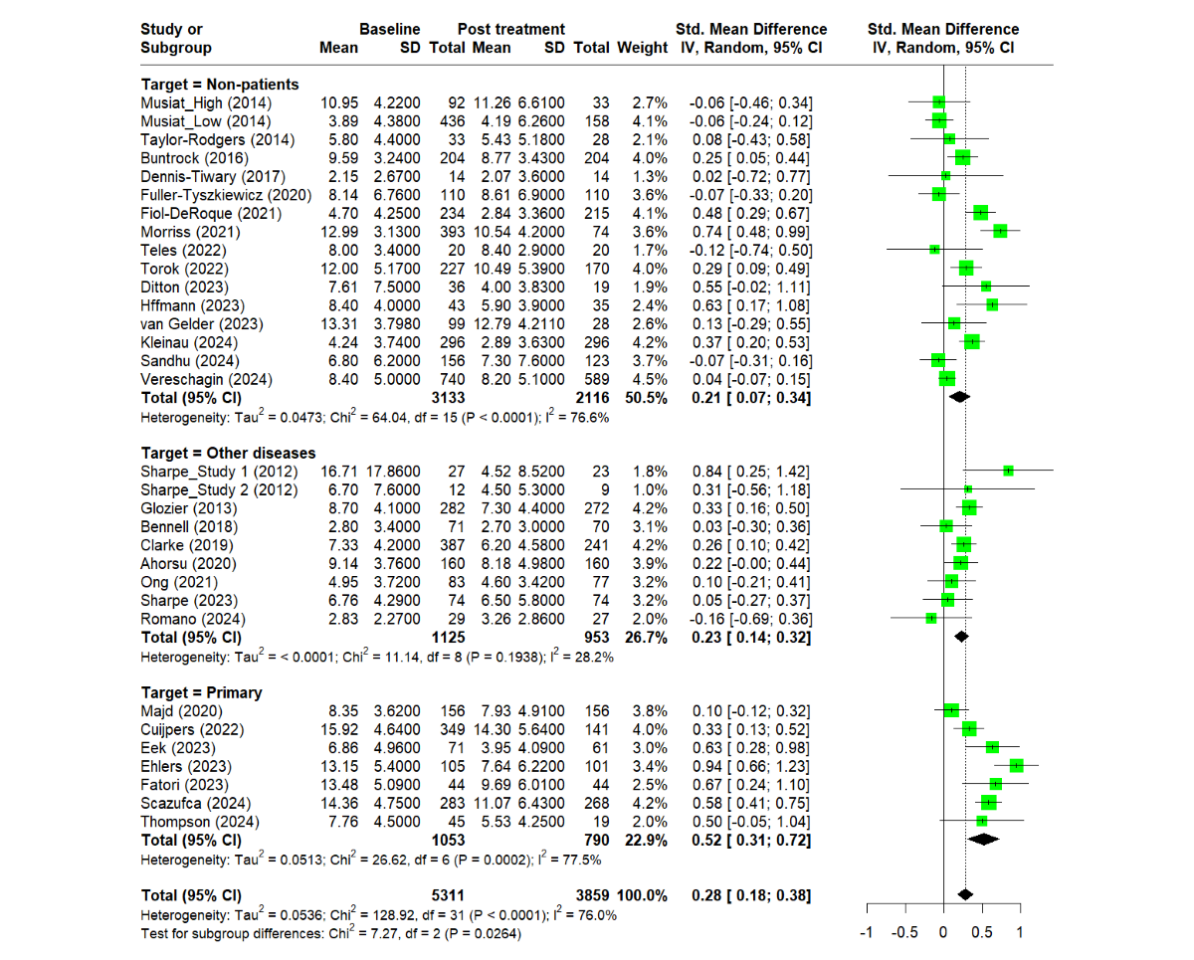
Forest plot of digital placebo effect from the subgroup analysis across primary psychiatric patients, patients with other diseases rather than psychiatric disorders and nonpatients. IV: inverse variance method; Other diseases: patients with other diseases rather than psychiatric disorders; Primary: primary psychiatric patients; Random: random effects model. Std: standardized [21,23,25,27,29,31,35,38,40,41,42,45,47,48,50,53,54,55,56,57,58,59,62,63,65,66,67,68,69,70].

The subgroup analysis across the 3 GAD assessment scores adopted in this study did not show the significant difference in the magnitude of placebo effect (*P*=.16). On the other hand, the subgroup analysis showed the significant difference in the magnitude of placebo effect across the target population (*P*=.03) although the placebo effect of all the subgroups was statistically significant (see Figure 3). The placebo effect of the subgroup for primary psychiatric disorders was larger than the one for the other 2 groups. The subgroup analysis also showed the significant difference in the magnitude among placebo approach (*P*=.02) and baseline value (*P*=.02). The placebo effect of the “Removed” type in placebo approach was smaller than other approaches. Also, the placebo effect was larger in the higher baseline groups in each GAD assessment score than the lower baseline groups.

We investigated the factors contributing to the high heterogeneity through meta-regression and identified that target population, placebo approach, baseline values, and GAD assessment scores as main contributing factors (*R*^2^=58.5%). The meta-regressions in this study also showed that the primary psychiatric patients in target population (*P*=.01), “Removed” type in placebo approach (*P*=.04) and high baseline values (*P*=.02) were statistically significant (see Table 2). There were no strong correlations between the 3 variables. The correlation coefficient between target population and baseline values was 0.15. The one between target population and placebo approach was 0.21. The one between baseline values and placebo approach was 0.09.

**Table 2.** Results of meta-regressions.

Variables	Level	N	Estimate	SE	*P* value	Lower limit	Upper limit
Publication year	N/A^a^	32	0.015	0.014	.28	− 0.012	0.041
Age	N/A	29	−0.0004	0.004	.93	−0.083	0.0076
Total number of patients	N/A	32	0.0000	0.0001	.89	−0.0003	0.0002
Treatment period	N/A	31	−0.001	0.001	.11	−0.002	0.0002
Number of groups	N/A	32	0.080	0.737	.46	−0.133	0.293
Target population	Nonpatients	32	Reference				
	Primary	—^a^	0.308	0.113	.01	0.087	0.530
	Other diseases	—	−0.005	0.108	.97	−0.216	0.206
Placebo delivery type	Mobile	32	Reference				
	Web	—	0.003	0.103	.97	−0.198	0.205
Placebo approach	Less	32	Reference				
	Removed	—	−0.048	0.240	.04	−0.954	- 0.013
	Replaced	—	−0.300	0.223	.18	−0.737	0.136
	Unrelated	—	−0.328	0.248	.19	−0.814	0.158
Blindness	Double	32	Reference				
	Single-investigators	—	0.032	0.118	.79	−0.200	0.264
	Single-patients	—	−0.161	0.118	.17	−0.392	0.070
Number of countries	Multiple	32	Reference				
	Single	—	−0.06	0.207	.77	−0.466	0.345
GAD^b^ assessment scores	DASS-A^c^	32	Reference				
	GAD-7^d^	—	0.172	0.114	.13	−0.050	0.395
	HADS-A^e^	—	0.005	0.138	.97	−0.266	0.276
Baseline values	High	32	Reference				
	Low	—	−0.222	0.094	.02	−0.406	−0.038

a N/A: not applicable.

b GAD: generalized anxiety disorder.

c DASS-A: Depression Anxiety Stress Scales-Anxiety.

d GAD-7: Generalized Anxiety Disorder-7.

e HADS-A: Hospital Anxiety and Depression Scale-Anxiety.

## Discussion

### Principal Findings

The systematic review in this study revealed that digital placebos have been actively adopted as comparators in recent DTx clinical trials and most of the study designs were parallel-group, blind RCTs conducted in a single country. The meta-analysis in our study revealed the small-to-moderate and statistically significant digital placebo effect on GAD assessment scores. The findings were not changed after the adjustment by trim-and-fill method, by risk of bias assessment, or by sensitivity analysis. The subgroup analyses also revealed the significant difference on the placebo effect across the target population, placebo approach, and baseline values. According to the meta-regressions, primary psychiatric disorders in target population, “Removed” type in placebo approach, and higher baseline values of GAD assessment scores were associated with effect of digital placebos. The correlation of each variable was not high and the 3 variables were considered to be independent moderators.

### Comparison With Previous Work

The finding that digital placebos have been actively adopted as comparators in clinical trials recently is consistent with the recent research [7]. The placebo approach adopted mostly was “replaced with inactive/neutral” in this study, which was consistent with the previous research that covered all the psychiatric disorders [8]. The placebo delivery type adopted mostly was web followed by mobile, and only 1 study adopted computer in this study, which was different from the previous research that computer, wearable, and virtual reality were adopted more [8]. With the advancement of digital technology, the ratio and variety of digital placebos used in clinical trials are expected to change in the future.

The study designs of the selected studies in this study were very similar to the ones with conventional pharmacotherapy. Most of them were parallel-group, blind RCTs. However, most of the clinical trials selected in this study were conducted in a single country, that was very different from the clinical trials of pharmacotherapy that MRCTs were quite common. The lack of internationally harmonized regulatory review and authorization system to facilitate the MRCTs is considered as one of the reasons in addition to the hurdles of language and sociocultural factors [77]..

To our knowledge, this study is the first research to investigate the magnitude of placebo effect and its moderators with meta-analysis. The result that the magnitude of the digital placebo effect in the group of primary psychiatric disorders was larger than the other 2 groups is consistent with a previous report that has shown the large placebo effect among GAD patients [17]. Further comprehensive research is needed to investigate the reasons the digital placebo effect in the group of primary psychiatric disorders was higher, but expectancy effects for digital devices and neurological changes observed in pharmacotherapy may occur even with digital placebos [78,79]. At the same time, the results in the group of primary psychiatric disorders in this study indicated that the magnitude of digital placebo effect might be smaller than the placebo effect in pharmacotherapy or neurostimulation [17,80]. Further research, which will compare the magnitude between digital placebos and conventional placebos with the same target population directly is needed to address this question.

In this study, “Removed” type in placebo approach was identified as a moderators of digital placebo effect. The result might be reasonable because the other 3 approaches still include less, unrelated, or neutral components of active interventions. The finding of higher baseline groups as a moderator might be also reasonable given the wide range of fluctuations. However, more research is needed to clarify whether these variables affect the magnitude of digital placebo effect.

### Limitations

There are several limitations in this study. First, we defined digital placebos based on previous research, under which no universal definition of digital placebos exists. The systematic review and meta-analysis with different definitions of digital placebos might lead to different conclusions. For example, the conclusion of meta-analysis may change if we include the cases where the delivery types between interventions and the controls were different. Second, this study used only 3 reliable academic databases used in medical and scientific research for the keyword search. The Egger test and funnel plot in this study suggested that publication bias was minimal. However, the risk of bias or incomplete evidence can not be ruled out. Third, this study included only adults aged 18 years and older. Other assessment scores need to be evaluated to investigate the digital placebo effect for children and adolescents. Fourth, we included only 3 GAD assessment scores, the reported number of which were the largest in this meta-analysis to control heterogeneity. These 3 assessment scores covered 74.9% (5311/7092) of all cases included in the systematic review, but more comprehensive studies are needed to discuss the generalizability of the findings in this study. Also, further studies on other diseases are needed to investigate the generalizability of the findings in this study beyond GAD. Fifth, we did not take the long-term effects of digital placebos into consideration. We adopted the postintervention data at the end of treatment or the closest to standardize the conditions although the long-term effect after the treatment was investigated in some selected studies. Finally, we did not consider the psychological or sociocultural factors that may impact on the effect of digital placebos.

### Conclusions

This study showed the small-to-moderate and statistically significant digital placebo effect on GAD assessment scores. Target population, the digital placebo approach, and high baseline values were also identified as moderators of the digital placebo effect in this study. It would be effective to create the study protocols for the DTx trials with digital placebos by considering the factors identified in this study.

## Supplementary material

10.2196/74905 Multimedia Appendix 1 Search strategy.

10.2196/74905 Multimedia Appendix 2 Characteristics of studies included in the systematic review.

10.2196/74905 Multimedia Appendix 3 Funnel plot and trim-and-fill funnel plot.

10.2196/74905 Multimedia Appendix 4 Results of risk of bias in each study.

10.2196/74905 Checklist 1 PRISMA (Preferred Reporting Items for Systematic Reviews and Meta-Analyses) 2020 checklist.

## Abbreviations

DASS-A: Depression Anxiety Stress Scales-Anxiety
DTx: digital therapeutics
GAD: generalized anxiety disorder
GAD-7: Generalized Anxiety Disorder-7
HADS-A: Hospital Anxiety and Depression Scale-Anxiety
MRCTs: multiregional clinical trials
PRISMA: Preferred Reporting Items for Systematic Reviews and Meta-Analyses
RCT: randomized controlled trial
SAD: social anxiety disorder
SaMD: software as a medical device
