# Supplementary material for: Magnitude of the Digital Placebo Effect and Its Moderators on Generalized Anxiety Symptoms: Systematic Review and Meta-Analysis
Source: J Med Internet Res. 2025 Jul 31;27:e74905. doi: 10.2196/74905 (PMC12337234; doi:10.2196/74905)
Supplement: Multimedia Appendix 1 [file jmir-v27-e74905-s001.docx]

Multimedia Appendix 2. Search Strategy

PubMed

("anxiety"[MeSH Terms] OR "anxiety"[All Fields] OR "anxieties"[All Fields] OR "anxiety s"[All Fields]) AND ("random*"[All Fields] OR "RCT"[All Fields]) AND ("blinded"[All Fields] OR "blinding"[All Fields] OR "blinds"[All Fields] OR "persons with visual disabilities"[MeSH Terms] OR ("persons"[All Fields] AND "visual"[All Fields] AND "disabilities"[All Fields]) OR "persons with visual disabilities"[All Fields] OR "blind"[All Fields] OR "blindness"[MeSH Terms] OR "blindness"[All Fields] OR ("blinded"[All Fields] OR "blinding"[All Fields] OR "blinds"[All Fields] OR "persons with visual disabilities"[MeSH Terms] OR ("persons"[All Fields] AND "visual"[All Fields] AND "disabilities"[All Fields]) OR "persons with visual disabilities"[All Fields] OR "blind"[All Fields] OR "blindness"[MeSH Terms] OR "blindness"[All Fields])) AND ("digitalisation"[All Fields] OR "digitalised"[All Fields] OR "digitalization"[All Fields] OR "digitalize"[All Fields] OR "digitalized"[All Fields] OR "digitalizer"[All Fields] OR "digitalizing"[All Fields] OR "digitally"[All Fields] OR "digitals"[All Fields] OR "digitization"[All Fields] OR "digitizations"[All Fields] OR "digitize"[All Fields] OR "digitized"[All Fields] OR "digitizer"[All Fields] OR "digitizers"[All Fields] OR "digitizes"[All Fields] OR "digitizing"[All Fields] OR "radiographic image enhancement"[MeSH Terms] OR ("radiographic"[All Fields] AND "image"[All Fields] AND "enhancement"[All Fields]) OR "radiographic image enhancement"[All Fields] OR "digital"[All Fields] OR ("mhealth s"[All Fields] OR "telemedicine"[MeSH Terms] OR "telemedicine"[All Fields] OR "mhealth"[All Fields]) OR ("telemedicine"[MeSH Terms] OR "telemedicine"[All Fields] OR "ehealth"[All Fields]) OR ("australas plant pathol"[Journal] OR "app"[All Fields]) OR ("ctsb protein human"[Supplementary Concept] OR "ctsb protein human"[All Fields] OR "apps"[All Fields]) OR "application*"[All Fields] OR ("smartphone"[MeSH Terms] OR "smartphone"[All Fields] OR "smartphones"[All Fields] OR "smartphone s"[All Fields]) OR ("mobile"[All Fields] OR "mobiles"[All Fields]) OR "online"[All Fields] OR "computer-based"[All Fields] OR "web-based"[All Fields] OR "internet-based"[All Fields] OR "internet-delivered"[All Fields] OR "virtual reality"[All Fields] OR ("vis resour"[Journal] OR "proc ieee virtual real conf"[Journal] OR "vr"[All Fields]) OR "augmented reality"[All Fields] OR ("anat rec a discov mol cell evol biol"[Journal] OR "adm radiol"[Journal] OR "adm radiol j"[Journal] OR "action res lond"[Journal] OR "ar"[All Fields]) OR "wearable*"[All Fields] OR "game*"[All Fields] OR "gamifi*"[All Fields])

Web of Science

ALL=((Anxiety AND (random* OR RCT) AND (blind OR blinded) AND (digital OR mhealth OR ehealth OR app OR apps OR application* OR smartphone OR mobile OR online OR computer-based OR web-based OR internet-based OR internet-delivered OR "virtual reality" OR VR OR "augmented reality" OR AR OR wearable* OR game* OR gamifi*)) )

Scopus

TITLE-ABS-KEY ( ( anxiety AND ( random* OR rct ) AND ( blind OR blinded ) AND ( digital OR mhealth OR ehealth OR app OR apps OR application* OR smartphone OR mobile OR online OR computer-based OR web-based OR internet-based OR internet-delivered OR "virtual reality" OR vr OR "augmented reality" OR ar OR wearable* OR game* OR gamifi* ) ) )
