# Supplementary material for: Magnitude of the Digital Placebo Effect and Its Moderators on Generalized Anxiety Symptoms: Systematic Review and Meta-Analysis
Source: J Med Internet Res. 2025 Jul 31;27:e74905. doi: 10.2196/74905 (PMC12337234; doi:10.2196/74905)
Supplement: Multimedia Appendix 2 [file jmir-v27-e74905-s002.docx]

Multimedia Appendix 3. Characteristics of studies included in systematic review

| Characteristics | n (%) | Characteristics | n (%) |
| --- | --- | --- | --- |
| **Publication year**  - 2014  　2015 - 2018  　2019 - 2022  　2023 - | 11 (18.5)  8 (16.7)  18 (33.3)  17 (31.5) | **Blindness**  　Single-Participants  　Single-Investigators  　Double | 16 (29.6)  13 (24.1)  25 (46.3) |
| **Target population**  　Primary psychiatric disorders  　Underlying with other diseases  　Non-patients | 21 (38.9)  14 (25.9)  19 (35.2) | **Treatment period (day)**  　 1 - 28  　29 - 56  　57 -  　Not specified (3-6 weeks) | 17 (31.5)  20 (37.0)  16 (29.6)  1 (1.9) |
| **Age**  　18 - 30  　31 - 40  　41 - 50  　51 - | 10 (18.5)  23 (42.6)  11 (20.4)  10 (18.5) | **Number of groups**  　2  　3  　4 - | 45 (83.3)  6 (11.1)  3 (5.6) |
| **Sham delivery type**  　Mobile  　Web  　Computer  　Virtual Reality | 16 (29.6)  34 (63.0)  1 (1.9)  3 (5.6) | **Number of countries**  　Single  　Multiple | 51 (94.4)  3 (5.6) |
| **Sham approach**  　Replaced with inactive/neutral  　Removed  　Replaced with unrelated  　Less intense | 38 (70.4)  7 (13.0)  6 (11.1)  3 (5.6) | **GAD assessment scores**  　GAD-7^a^  　DASS-A^b^  　HADS-A^c^  　BAI^d^  　STAI-ST^e^  　HAM-A^f^  　PSWQ^g^  　STAI-TR^h^  　GADQ^i^  　HADS^j^  　PROMIS-A^k^  　STAI^l^ | 14 (25.9)  11 (20.3)  7 (14.8)  6 (11.1)  4 (7.4)  3 (5.6)  3 (5.6)  2 (3.7)  1 (1.9)  1 (1.9)  1 (1.9)  1 (1.9) |
| **Total number of participants**  - 50  51 - 250  　251 - 500  　501 - | 9 (16.7)  28 (51.9)  9 (16.7)  8 (14.8) |  |  |

^a^GAD-7: Generalized Anxiety Disorder-7

^b^DASS-A: Depression Anxiety Stress Scales-Anxiety

^c^HADS-A: Hospital Anxiety and Depression Scale-Anxiety

^d^BAI: Beck Anxiety Inventory

^e^STAI-ST: State Trait Anxiety Inventory-State

^f^HAM-A: Hamilton Anxiety Rating Scale

^g^PSWQ: Penn State Worry Questionnaire

^h^STAI-TR: State Trait Anxiety Inventory-Trait

^i^GADQ: Generalized Anxiety Disorder Questionnaire

^j^HADS: Hospital Anxiety and Depression Scale

^k^PROMIS-A: Patient-Reported Outcomes Measurement Information System-Anxiety

^l^STAI: State Trait Anxiety Inventory
