# Supplementary material for: Magnitude of the Digital Placebo Effect and Its Moderators on Generalized Anxiety Symptoms: Systematic Review and Meta-Analysis
Source: J Med Internet Res. 2025 Jul 31;27:e74905. doi: 10.2196/74905 (PMC12337234; doi:10.2196/74905)

Multimedia Appendix 4. Funnel plot and Trim-and-fill funnel plot.

Funnel Plot


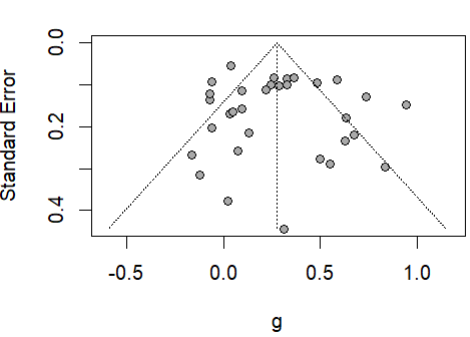


Trim-and-fill funnel plots. Gray dots and white dots indicate the selected studies and the imputed studies, respectively.


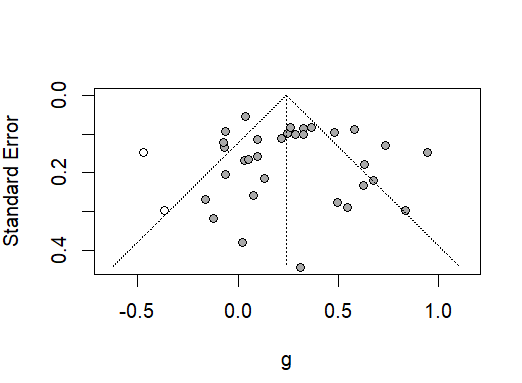

Supplement: Multimedia Appendix 3 [file jmir-v27-e74905-s003.docx]
