# Supplementary material for: Magnitude of the Digital Placebo Effect and Its Moderators on Generalized Anxiety Symptoms: Systematic Review and Meta-Analysis
Source: J Med Internet Res. 2025 Jul 31;27:e74905. doi: 10.2196/74905 (PMC12337234; doi:10.2196/74905)
Supplement: Multimedia Appendix 4 [file jmir-v27-e74905-s004.docx]

Multimedia Appendix 5. Results of risk of bias in each study.
